# Mental Health and Family Functioning in Patients and Their Family Members after Traumatic Brain Injury: A Cross-Sectional Study

**DOI:** 10.3390/brainsci10100670

**Published:** 2020-09-25

**Authors:** Mari S. Rasmussen, Juan Carlos Arango-Lasprilla, Nada Andelic, Tonje H. Nordenmark, Helene L. Soberg

**Affiliations:** 1 Department of Physical Medicine and Rehabilitation, Oslo University Hospital, P.B. 4950 Nydalen, 0424 Oslo, Norway; nadand@ous-hf.no (N.A.); tofoss@ous-hf.no (T.H.N.); h.l.soberg@medisin.uio.no (H.L.S.); 2 Institute of Health and Society, Research Centre for Habilitation and Rehabilitation Models & Services (CHARM), Faculty of Medicine, University of Oslo, 0318 Oslo, Norway; 3 Biocruces Bizkaia Health Research Institute, 48903 Barakaldo, Spain; jcalasprilla@gmail.com; 4 IKERBASQUE, Basque Foundation for Science, 48009 Bilbao, Spain; 5 Department of Cell Biology and Histology, University of the Basque Country UPV/EHU, 48940 Leioa, Spain; 6 Department of Psychology, University of Oslo, 0317 Oslo, Norway; 7 Department of Physiotherapy, Faculty of Health Sciences, OsloMet—Oslo Metropolitan University, 0130 Oslo, Norway

**Keywords:** traumatic brain injury, quality of life, family functioning, rehabilitation

## Abstract

Traumatic brain injury (TBI) affects the family as a whole. This study aimed to describe and compare mental health and family functioning in TBI patients and their family members, and to identify individual and family-related factors that were associated with mental health. It was conducted at an urban, specialized, TBI outpatient clinic and included 61 patients with mild to severe TBI and 63 family members. Baseline demographics and injury-related data were collected, and the participants answered standardized, self-reported questionnaires 6–18 months post-injury that assessed mental health; general health; family functioning, communication, and satisfaction; depression and anxiety; self-efficacy; resilience; and condition-specific quality of life. The patients reported significantly worse mental health, depression, resilience, self-efficacy, and general health compared with the family members. Patients and family members had similar perceptions, showing balanced family functioning, high family communication levels, and moderate family satisfaction. Factors significantly associated with mental health in patients and family members were depression, anxiety, and resilience, explaining 56% of the variance (*p* < 0.001). Family-related factors were not associated with mental health. The disease burden was mainly on the patients; however, the family members also reported emotional distress. Family-targeted interventions across the TBI continuum should be considered.

## 1. Introduction

The consequences of traumatic brain injury (TBI) are multifaceted and affect the patients’ family as well [1,2]. Persistent physical, cognitive, and emotional problems have been identified in individuals with TBI of all severities [3]. A previous study demonstrated that up to 20% of individuals with TBI of all severity levels experienced symptoms, such as dizziness, fatigue, headaches, depression, and anxiety, one year after the injury [4]. TBI adversely affects health-related quality of life (HRQL) across both the mental and physical domains [5], and HRQL is as an important outcome in populations with TBI of all severity levels [6].

When patients return home after the primary rehabilitation period, they often rely on their family members for emotional and practical support in everyday life activities [7]. The family members consequently have a fundamental role in the patients’ rehabilitation. The changes in lifestyle and responsibilities of the family members are challenging, and an increased caregiver burden [8,9,10], diminished HRQL [11,12], and increased levels of emotional distress [13,14] are negative outcomes for the caregivers.

Families are not prepared for the sudden changes caused by TBI; therefore, they are at risk for disrupted family dynamics that may lead to unhealthy family functioning. Researchers have documented a significant and lasting increase in unhealthy family functioning following TBI [15,16]. The stress on the family system seems to be less dependent on the severity of the TBI, but is related to the cognitive, emotional, and behavioral changes in the injured person [17,18]; and there is a reciprocal relationship among TBI-related factors in the injured person, caregiver distress, and family functioning [14,16,19].

To form a more comprehensive picture of the challenges that families face after TBI, it is important to include the perspectives of both the patients and their family members [20]. Previous research has demonstrated that personal, familial, and social characteristics and injury severity affect HRQL, disease burden, and family adjustment after TBI [21,22,23]. However, there is a paucity of knowledge on mental health and family functioning related to the consequences of TBI, which include the impact on these outcomes for members within the same family system. Thus, the objectives of this study were to:

(a) Describe and compare aspects of mental health and family functioning in home-dwelling patients with TBI and their family members at 6–18 months post-injury (i.e., the study inclusion time)
(b) Explore individual- and family functioning-related factors that are associated with mental health.

## 2. Materials and Methods

### 2.1. Study Design and Settings

Data were collected from June 2017 to June 2019 at Oslo University Hospital (OUH) in Norway as part of a two-armed, pragmatic, randomized, controlled trial (RCT) aimed at evaluating the effectiveness of a family intervention called the “Traumatic Brain Injury/Spinal Cord Injury Family Intervention” [24]. The intervention was originally developed based on experiences from TBI rehabilitation services in Latin America. Adaptations of the intervention into a Norwegian setting have been published by our group [25]. This study was approved by the Regional Committee for Medical and Health Research Ethics, South-East Norway (approval no. 2016/1215), and the Data Protection Officer at OUH. It was registered at ClinicalTrials.gov with the identification number NCT03000400. The study presented cross-sectional data from the first assessment time point (T1), which we defined as the baseline assessment of the RCT.

### 2.2. Participants

A total of 251 patients with TBI were approached at the TBI Outpatient Clinic of OUH for participation. Patients are generally referred to this outpatient clinic for follow-ups with a physiatrist and, if needed, with a multiprofessional rehabilitation team consisting of medical doctors, a psychologist, physical therapists, occupational therapists, and a social worker. The patients either were inpatients at the hospital in the acute phase or were referred to the outpatient clinic by their general practitioner. The patients referred to the outpatient clinic with mild TBI had experienced persistent post-concussion symptom pressure. All the patients were evaluated by a physiatrist and were considered eligible based on the following inclusion and exclusion criteria: (a) age between 16 and 65 years; (b) diagnosed with TBI of any severity according to the International Classification of Diseases (ICD)-10 classification system (S06.0–S06.9); (c) a Rancho Los Amigos Revised Scale score of 8 [26]; (d) TBI sustained 6 to 18 months ago; and (e) were home dwelling. The family members were chosen by the patients and considered eligible if they: (a) were between 18 and 65 years and (b) were actively involved in the patients’ daily life with weekly contact. The exclusion criteria that applied to all the eligible participants were: (a) inability to speak/read Norwegian; (b) a pre-injury learning disability; (c) an ICD-10 diagnosis of severe psychiatric or degenerative neurological illness; (d) ongoing substance abuse; and (e) families in which other family members required professional care. Eligible patients and family members received oral and written information regarding the study. All participants provided written informed consent.

### 2.3. Measures

The following sociodemographic characteristics were recorded: participant role (patient/family member), age (years), sex (female/male), marital status (married/partner/single), length of relationship (<1 year/1–5 years/>5 years), education (dichotomized as low/high with high representing college/university degree), and current work status (full-time work/partial sick-leave/sick-leave). From the patients only, we collected pre-injury work status (not working/working), comorbidities (no/yes), and number of persons living in the household. The following data were collected from the family members: type of relationship with the injured individual (spouse/parent/child) and whether they lived in the same household as the injured individual (no/yes).

The patients’ injury-related characteristics included time since injury (in weeks); injury mechanism (fall/traffic accident/mechanical object/violence/others); neuroimaging results of intracranial injury (no/yes); length of hospital stay (days); the lowest Glasgow Coma Scale (GCS) score recorded within the first 24 h after the injury, with scores from 3–8 indicating severe TBI, 9–12 indicating moderate TBI, and 13–15 indicating mild TBI [27]; the Abbreviated Injury Scale (AIS)—head score that is calculated as a standardized approach for categorizing the type and severity of injuries to the head [28]; and post-concussion symptoms assessed with the Rivermead Post-Concussion Symptoms Questionnaire (RPQ) [29]. The RPQ has a scale with scores ranging from 0 (best) to 64 (worst), and the total score was used in the present study. The RPQ has been validated in Norway [30].

HRQL measure: The primary outcome measure was the 36-item Short Form Health Survey (SF-36) Mental Component Summary (MCS) score [31]. The MCS was aggregated from the four mental health scales that are part of the SF-36, Vitality (VT), Social Functioning (SF), Role Limitation Due to Emotional Problems (RE), and Mental Health (MH) scales, as the weighted sum of the subscale scores with a mean value of 50 and a standard deviation of 10. The General Health Scale (GH) of the SF-36, which provides an overall evaluation of the health status, was applied. The MCS and GH each have scores ranging from 0 (worst) to 100 (best). Scores below 40 indicate impaired mental health/general health [32]. The SF-36 has demonstrated good validity, reliability, and responsiveness in TBI populations with Cronbach’s alphas ranging from 0.79 to 0.92 [31].

Family functioning measure: The Family Adaptability and Cohesion Evaluation Scale, fourth edition (FACES IV) [33], is a 42-item scale consisting of two balanced scales (flexibility and cohesion) and four unbalanced scales (disengaged, chaos, enmeshed, and rigid). The scales are used to determine the level of flexibility and cohesion within couples and family systems and include a circumplex ratio score ranging from 0 (worst) to 10 (best) that is assigned to the level of cohesion and flexibility within a family; a score of 1 indicates equal amounts of balance and unbalance in the family system. In addition, the Family Communication Scale (FCS) that assesses communication skills within the family and the Family Satisfaction Scale (FSS) that assesses the level of satisfaction with family functioning were used. The raw scores of each scale were recoded into percentile scores ranging from 10 (worst) to 99 (best). FACES IV is proven valid and reliable with Cronbach’s alphas ranging from 0.77 to 0.93 [33,34].

Psychological functioning: Depression was measured with the Patient Health Questionnaire-9 (PHQ-9), which is a nine-item screening instrument used to evaluate the severity of symptoms of depression [35]. The scores can be interpreted as follows: no depression (0–4 points), mild (5–9 points), moderate (10–14 points), moderately severe (15–19 points), and severe depression (20–27 points). The psychometric properties of the scale are favorable [35].

Anxiety was measured with the Generalized Anxiety Disorder Questionnaire-7 (GAD-7). This is a seven-item questionnaire that is used to assess symptoms of generalized anxiety [36]. The scores can be interpreted as follows: mild (5–9 points), moderate (10–14 points), and severe anxiety (15–21 points). The GAD-7 has demonstrated excellent construct validity with a Cronbach’s alpha of 0.92 [36].

Self-efficacy: The General Self-Efficacy Scale (GSE), which assesses the belief in a person’s own competence to handle stressful events and demands, was used [37]. It has 10 items with an ordinal scale from 1 (not at all true) to 4 (exactly true), and scores ranging from 10 (worst) to 40 (best). High reliability and construct validity on this scale have been confirmed in earlier studies, with Cronbach’s alpha values of 0.86–0.94 [37].

Resilience: The Resilience Scale for Adults (RSA) was used to assess protective factors in individuals [38]. This 33-item scale covers five dimensions (1A, perception of self; 1B, perception of future; 2, social competence; 3, family cohesion; 4, social resources; and 5, structured style). The scale’s scores range from 0 (worst) to 165 (best). The total score was used in this study. The RSA has demonstrated adequate internal consistency with Cronbach’s alphas ranging from 0.76 to 0.87 [38].

Condition-specific quality of life measure: The Quality of Life after Brain Injury Questionnaire (QOLIBRI) is a 37-item scale consisting of six subscales that include four satisfaction scales (the Cognition, Self, Daily Life, and Autonomy scales) and two bothered scales (the Emotions and Physical Problems scales) [39]. A total score ranging from 0 (worst) to 100 (best) can be calculated. A score <60 points indicates a reduced quality of life [40]. The Norwegian QOLIBRI has exhibited satisfactory psychometric properties with Cronbach’s alphas ranging from 0.75 to 0.96 [41].

### 2.4. Data Sources

The sociodemographic data were obtained from a self-reported questionnaire developed by the authors (M.S.R. and H.L.S.), whereas the injury-related variables were collected from the patients’ medical records. For seven patients, the GCS score was not specified in the medical records and was assigned by a physiatrist (author N.A.) based on the injury descriptions in the patients’ medical records. At the study’s baseline assessment, all the self-reported outcome measures were filled out after the family had been allocated to the intervention group or the control group in the RCT.

### 2.5. Study Sample Size

Sample size estimation was based on the primary outcome measure, SF-36 MCS. This was based on a study on Norwegian patients with moderate to severe TBI [42]. An estimated sample size of 66 patients was calculated with α = 0.05 and β = 0.2, taking into consideration a 10% dropout rate.

### 2.6. Data Analysis and Statistics

All the statistical analyses were performed using IBM SPSS Statistics 25. The distribution of the data was evaluated by using the Kolmogorov–test and/or visual plots. The mean and standard deviation (SD) were used for normally distributed data, and the median and interquartile range (IQR) were used for skewed data. Categorical data are presented as frequencies and percentages.

Missing data were addressed by replacing the missing scores on a scale or subscale with the mean score of the remaining variables if appropriate. Missing data points in the SF-36 were automatically handled by the scoring software program, PRO CoRE 1.5 Smart Measurement System (Optum, Eden Prairie, MN, USA). The data were checked for internal dependency within each family using an intraclass correlation coefficient (ICC), and evaluated as poor with an ICC value of 0.107 [43]. Differences between patients and family members were evaluated by using independent *t*-tests or Mann–Whitney *U* tests. The chi-square test was used to detect group differences in categorical data. Statistical significance was determined by a *p*-value of <0.05.

MCS was the dependent variable in the multiple regression analysis. Univariate linear regressions were used to evaluate associations between the independent variables and MCS. Independent variables with a *p*-value ≤0.1 were selected and included in the multiple regression analysis. Age did not fulfill the inclusion criteria of a *p*-value of ≤0.1, but was included in the multiple regression analysis to adjust for variations in the population. Correlations between independent variables were evaluated by performing Spearman’s Rho test, and variables with an intercorrelation >0.7 were not applied together in the multiple regression analysis. These analyses are not presented. Owing to a the intercorrelation between FCS and FSS with a Spearman’s rho >0.7, the FCS score was chosen as the candidate variable in the multiple regression analysis because family communication was at the core of this study and can be seen as a facilitator for family functioning [33].

One hundred twenty-two participants were included in the regression analysis because two respondents were excluded on account of missing data, and we identified a maximum of 10 independent variables to be included in the analysis [44]. A multiple linear regression analysis with a backward approach was conducted to assess the associations among personal factors, individual functioning, and family functioning.

Prior to carrying out the multiple regression analyses, we investigated the possibility of multicollinearity among the independent variables using a variance inflation factor. We controlled for the normality of residuals by inspecting the histogram and quantile-quantile plots. To check for internal validity, the model was run with 1000 bootstrap samples [44]. The results of the multiple regression modeling are presented with R^2^, adjusted R^2^, and unstandardized B coefficients with 95% confidence intervals.

## 3. Results

**Figure 1:**
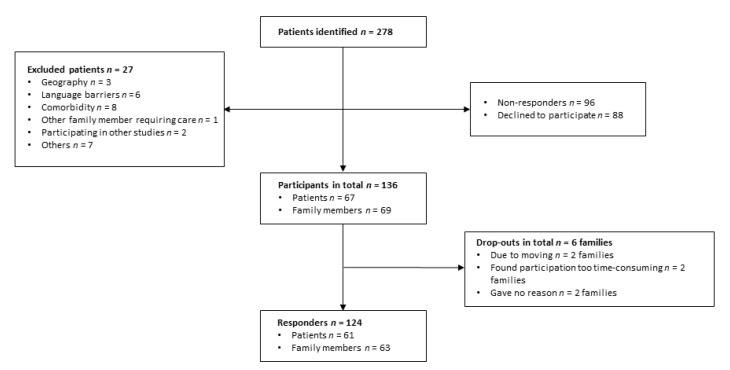
Flow chart.

In total, 67 patients and 69 family members agreed to participate in this study. Six families withdrew after the randomization for the following reasons: two families moved away, two families thought participating would be too time consuming, and two families did not provide a specific reason, leaving a total of 124 participants at baseline. See Figure 1 for flow chart.

Table 1 presents the sociodemographic data. Baseline data were collected from 61 patients (54.1% women) with a mean (SD) age of 43.8 (11.2) years and from 63 family members (52.4% women) with a mean (SD) age of 42.6 (11.3) years. There were no significant differences in sex, age, and level of education between the patients and family members. The majority of family members (92%) was spouse/partner of the injured person and more than 80% had been in the relationship for >5 years.

### Injury Characteristics

The injury characteristics are presented in Table 2. The median time since injury was 49 (IQR 36, 69) weeks. The median GCS score was 15 (IQR 14, 15), which corresponds to mild TBI. The median AIS head score of 1 (IQR 1, 3) indicated a mild severity level of injury. Among the 50 (82%) patients who had mild TBI as assessed by the GCS, eight (16%) patients had intracranial injury diagnosed with computed tomography/magnetic resonance imaging (CT/MRI) and were classified as having complicated mild TBI [45]. The main injury mechanisms were falls (37.7%) and traffic accidents (31.1%). The mean RPQ total score of 27.7 (SD 11.1) indicated a higher level of post-concussive symptoms.

At baseline, the patients reported significantly worse scores than the family members on overall mental health on the MCS, general health (GH) (SF-36), depression (PHQ-9), resilience (RSA), and self-efficacy (GSE) (Table 3). On the MCS, the difference was 5.9 points (*p* = 0.001). Overall, approximately one-third of the participants had impaired MCS with scores <40 points.

Regarding self-reported symptoms of depression on the PHQ-9, 65% of the patients reported scores that indicated mild to moderate depression and 20% reported scores indicating moderately severe to severe depression. Among the family members, 57% reported scores indicating mild to moderate depression and 6% reported scores indicating moderately severe to severe depression. The patients’ GAD-7 mean score indicated mild anxiety, whereas the family members’ GAD-7 mean score was just below the cut-off score for mild anxiety. Furthermore, 66.7% of the patients reported good GH with scores ≥40 points, whereas 90.5% of the family members reported good GH. The patients’ mean (SD) total QOLIBRI score was 58.1 (16.1) points, indicating diminished HRQL.

There were no statistical differences in terms of family functioning, communication, or satisfaction at baseline. Overall, the participants perceived their family functioning as balanced with a mean (SD) circumplex ratio score of 3.0 (1.1) for the patients and 3.1 (1.2) for the family members. On average, both the patients and their family members reported having high family communication levels (FCS) and a moderate level of family satisfaction (FSS).

The results of the univariate regression analyses of candidate variables in the multiple regression model that examined factors associated with MCS and the results of the multiple regression analysis are presented in Table 4.

In the final regression model, sex was the only personal factor that was significantly associated with the MCS, with men having 2.6-point higher scores than women. The individual functioning-related factors that were associated with the MCS were depression on the PHQ-9, anxiety on the GAD-7, and resilience on the RSA, whereas self-efficacy on the GSE and GH on the SF-36 were not associated with the MCS. The family functioning circumplex ratio and FCS scores were not significantly associated with the MCS. The final model accounted for 56.2% of the variance in the MCS (*p* < 0.001).

## 4. Discussion

In the present study, we focused on aspects of mental health and family functioning in both patients and family members after TBI as part of the baseline assessment of an ongoing RCT. Most patients had mild TBI with persistent symptoms and functional disturbances, and the symptom pressure as assessed with the RPQ at the time of inclusion was higher than that reported in previous studies on mild TBI [4,30].

Both sexes were equally represented, and being male was associated with better mental health in both patients and family members. This finding is in accordance with studies on mental health on the Norwegian general population [46], and with studies on TBI patients, in which women tended to report lower mental health scores and HRQL [5,42]. In addition, previous studies have demonstrated that women caregivers report lower quality of life levels compared with men [47,48].

### 4.1. Post-Injury Functioning

The patients in the current study reported significantly lower mental health levels compared with the family members; 48% of the patients and 17% of the family members reported poor mental health with MCS scores <40.0 points. A systematic review on HRQL after TBI found that mental HRQL (MCS) was more negatively affected than physical HRQL (PCS) [6]. The patients in this study also reported reduced HRQL as assessed by the QOLIBRI, which is considered to be more sensitive in terms of capturing specific domains often affected by TBI [49].

In previous studies, caregivers of individuals with TBI reported diminished HRQL compared with the general population [11,12]. However, only a few studies emphasized on patients with mild TBI, as in the present study. It is possible that the family members’ mental health may have been less affected because they did not have the same caregiver responsibilities or role changes as those of patients with more severe injuries as reported in the PariS-TBI study (47.7 points vs. 36.7 points) [50]. A study on caregivers of persons with mild TBI demonstrated that, at 6 months after the injury, the HRQL reached levels similar to the general population, suggesting that mild TBI has less impact on the family members’ mental health compared with more severe injuries [51]. We included family members 6–18 months post-injury supporting this finding on overall mental health; however, this impression was not clear-cut.

Symptoms of depression and anxiety are often described in research on individuals with TBI [52] and caregivers [13]. In the current study, the patients reported significantly worse depressive symptoms than the family members, which is in line with other studies [17,18]. Worse functional status in the injured person and need for supervision have previously been identified as risk factors for caregiver depression [53]. In spite of a possibility of less caregiver challenges than is the case with severe TBI, our results revealed that more than half of the family members experienced some level of emotional distress. Stevens et al. demonstrated that the caregivers’ perception of patient depression was the best predictor for depression in caregivers [54].

Based on the regression analysis, depression and anxiety were the two factors that were most strongly associated with mental health on the MCS. Studies have demonstrated that depression negatively affects HRQL in individuals with TBI [42,55,56]. Additionally, in caregivers, improvements in HRQL have been associated with improvements in symptoms of depression and anxiety [57], and strong correlations between depression and HRQL have been identified [58]. Although much of this research refers to patients and caregivers facing moderate to severe TBI, parallel associations were found in our study.

Compared with the patients, the family members reported significantly higher resilience and general self-efficacy. However, when controlled for other factors, only resilience contributed significantly to the variance in mental health, and higher resilience levels were associated with better mental health. Similarly, studies have shown that resilience in caregivers affects hope, which is positively associated with mental health and quality of life, and negatively associated with emotional distress [59,60]. Furthermore, patients with mild to severe TBI have been found to report lower resilience compared with the general population [61], and higher resilience is associated with fewer PCS symptoms after mild TBI [62].

### 4.2. Family Functioning

Healthy family functioning has been associated with better outcomes for TBI patients [63], and family functioning has shown to be positively associated with the mental health of patients and especially, the caregivers [20,64]. However, in our study, family functioning and communication were not significantly associated with overall mental health when controlled for other factors. On average, the patients and family members had similar perceptions, which showed balanced family functioning, high levels of family communication, and moderate family satisfaction. Even though research shows an increase in unhealthy family functioning after TBI [15,16], our results are in line with those of a study on families living in Latin America using the FACES IV data. The study demonstrated that a large proportion of the families had balanced levels of cohesion and flexibility as well as a high level of communication [65].

In the Latin American study, the authors argued that divergent results on family functioning across countries might be due to differences in family cultures, i.e., the Latin American family culture being characterized by family loyalty and placing the family needs above individual needs [65]. By contrast, families in the western societies have traditionally been characterized by relatively weak family links, where much of the support for family members have been provided by public and private institutions [66]. Thus, there might be a selection bias of families in the current study. It is conceivable that families who perceive their family communication as good would be more willing to participate in a study potentially involving participation in a family intervention. Families who were more troubled prior to the TBI might find it too challenging to address family problems with the extra strain that TBI might have imposed on the family system as different psychological, financial, and social factors play a role in a couple’s willingness to participate in research [67].

Moreover, the patients had undergone follow-up examinations and rehabilitation at a specialized outpatient clinic before being included in the current study. Norway functions as a welfare state that guarantees all its inhabitants approximately the same access to health services. The study was conducted in an urban area, and people living in urban areas are more likely to have contact with specialist physician services compared with people in rural areas [68]. This might explain why the level of strain on family functioning seemed lower in this study compared with studies conducted in countries with limited access to rehabilitation services. Ponsford et al. found that families who had access to comprehensive rehabilitation services after mild to severe TBI on average showed healthy family adjustment after the injury [18].

The current study has some limitations that should be considered. First, the data presented were collected at only one time point, and causality cannot be inferred. Second, the outcomes in the current study were based on self-reported measures; thus, the patients were only screened for depression and anxiety. Third, we did not specifically measure fatigue, which might be associated with mental health after TBI; however, the SF-36 vitality subscale is included in the MCS. Fourth, caution should be applied when generalizing the results of this study to families with moderate and severe TBI only because most of the participants in this study had mild TBI.

A few studies have reported on mental health and family functioning from the perspective of members within the same family system. A strength of this study is that it provides valuable insights into how TBI affects both patients and their closest family members within the same family unit by treating the family as a whole. Finally, this mixed sample of individuals with TBI represented the patients that were admitted to our department, which makes it possible to translate the findings to everyday clinical practice owing to increased external validity.

## 5. Conclusions

This study demonstrated that TBI, regardless of injury severity level, has lasting consequences on overall mental health and emotional functioning for the patients, but also to some extent for the family members. The disease burden was primarily on the patients as they had significantly lower mental health, overall general health, resilience, self-efficacy, and worse symptoms of depression compared with their family members. The family members reported some symptoms of depression. The participants had similar perceptions showing balanced family functioning. Family communication was perceived as high and family satisfaction as moderate. Sex, depression, anxiety, and resilience were significantly associated with mental health on the MCS, and family-related factors were not associated with the MCS in the regression analysis. Future studies assessing the effectiveness of targeted family interventions aimed at improving mental health in patients with TBI, especially those with a protracted course of recovery, and their family members should be considered.

## Figures and Tables

**Table 1:** Demographic factors of the 61 patients and 63 family members. SD, standard deviation.

	Patients (*n* = 61)		Family Members (*n* = 63)	
	Frequency (%)	Mean (SD)	Frequency (%)	Mean (SD)
Age, mean (SD)		43.8 (11.2)		42.6 (11.3)
Sex (% females)	33 (54.1)		33 (52.4)	
Comorbidity	12 (19.6)			
Marital status				
Married	35 (57.4)		37 (58.7)	
Partner/cohabitant	24 (39.3)		24 (38.1)	
Single	2 (3.3)		2 (3.2)	
Length of relationship				
<1 year	3 (5.0)		3 (4.9)	
1–5 years	7 (11.9)		8 (13.1)	
>5 years	49 (83.1)		50 (82.0)	
Living arrangement				
Living in the same household as the patient			57 (90.5)	
Number of people living in the patient’s household		3.1 (1.2)		
Education				
Low	16 (26.2)		15 (23.8)	
High	45 (73.8)		48 (76.2)	
Patients’ pre-injury work status				
Not working	4 (6.6)			
Working	57 (93.4)			
Current work status				
Full-time work	5 (8.2)		53 (84.1)	
Partial sick-leave	33 (54.1)		4 (6.3)	
Sick-leave	23 (37.7)		6 (9.5)	
Type of relation to the injured				
Partner/spouse			58 (92.1)	
Parent			1 (1.6)	
Child			4 (6.3)	

**Table 2:** Injury characteristics.

Injury Characteristics (*n* = 61)	Frequency (%)	Mean (SD)/Median (IQR)
Glasgow Coma Scale score		15 (14, 15)
Mild TBI	50 (82.0)	
Moderate TBI	3 (4.9)	
Severe TBI	8 (13.1)	
AIS head score		1.0 (1, 3)
Intracranial injury	18 (29.5)	
Surgical procedure	8 (13.1)	
Falls	23 (37.7)	
Traffic accidents	19 (31.1)	
Mechanical object	14 (23.0)	
Violence/Assault	2 (3.3)	
Others	3 (4.9)	
Time since injury (weeks)		49.4 (36, 69)
Length of stay (days)		5.4 (range 0–37)
RPQ total score (*n* = 56)		27.7 (11.1)
Self-reported comorbidities (*n* = 59)	11 (18.6%)	

TBI, traumatic brain injury; AIS, Abbreviated Injury Scale; RPQ, Rivermead Post-Concussion Symptoms Questionnaire; SD, standard deviation; IQR, interquartile range.

**Table 3:** Intergroup differences on self-reported outcome measures.

Outcome	Patients	Family Members		
	Mean (SD)	Mean (SD)	Mean difference	*p*-Values
SF-36 MCS	41.8 (9.9)	47.7 (9.0)	5.9	0.001
GH (SF-36)	45.8 (10.6)	54.4 (10.0)	8.6	<0.001
QOLIBRI overall scale	58.1 (16.1)	-	-	-
FACES IV circumplex ratio	3.0 (1.1)	3.1 (1.2)	0.1	0.692
FCS	65.7 (26.3)	66.4 (25.0)	0.7	0.884
FSS	55.6 (28.7)	55.3 (26.4)	0.3	0.946
PHQ-9	9.6 (5.1)	6.3 (4.3)	3.3	<0.001
GAD-7	6.0 (4.2)	4.9 (3.7)	1.1	0.193
RSA	107.2 (16.6)	113.9 (16.2)	6.7	0.025
GSE	30.1 (5.1)	31.8 (4.5)	1.7	0.044

SF-36, short form-36; MCS, Mental Component Summary; QOLIBRI, Quality of Life after Brain Injury Questionnaire; FACES IV, Family Adaptability and Cohesion Evaluation Scale; FCS, Family Communication Scale; FSS, Family Satisfaction Scale; PHQ-9, Patient Health Questionnaire-9; GAD-7, Generalized Anxiety Questionnaire-7; RSA, Resilience Scale for Adults; GSE, General Self-Efficacy Scale; GH, General Health; SD, standard deviation.

**Table 4:** Results of the regression analyses and results of the final multiple regression model.

Outcome Variable MCS	Univariate Regression		Multiple Regression Backward		
	B (C.I.)	*p*-Value	B	*p*-Value	95% C.I.
Age	0.09 (−0.68, 0.25)	0.265			
Sex (female/male)	3.09 (−0.42, 6.58)	0.084	2.56	0.038	(0.14, 5.0)
Relation (patient/family)	5.85 (2.47, 9.23)	0.001			
FACES IV circumplex ratio	2.37 (0.91, 3.84)	0.002			
FCS	0.08 (0.02, 0.15)	0.018			
FSS	0.11 (0.05, 0.17)	<0.001			
PHQ-9	−1.41 (−1.67, −1.16)	<0.001	−0.79	<0.001	(−1.16, −0.43)
GAD-7	−1.59 (−1.94, −1.25)	<0.001	−0.64	0.003	(−1.06, −0.22)
RSA	0.32 (0.23, 0.41)	<0.001	0.12	0.007	(0.04, 0.21)
GSE	0.96 (0.63, 1.28)	<0.001			
GH (SF-36)	0.43 (0.29, 0.57)	<0.001			
R^2^			0.576		
Adjusted R^2^			0.562		
F value			39.76	<0.001	

SF-36, short form-36; MCS, Mental Component Summary; FACES IV, Family Adaptability and Cohesion Evaluation Scale, fourth edition; FCS, Family Communication Scale; FSS, Family Satisfaction Scale; PHQ-9, Patient Health Questionnaire-9; GAD-7, Generalized Anxiety Questionnaire-7; RSA, Resilience Scale for Adults; GSE, General Self-Efficacy Scale; GH (SF-36), General Health; C.I., confidence interval.
